# Zacopride stimulates 5-HT_4_ serotonin receptors in the human atrium

**DOI:** 10.1007/s00210-024-03051-5

**Published:** 2024-04-01

**Authors:** Joachim Neumann, Christin Hesse, Britt Hofmann, Ulrich Gergs

**Affiliations:** 1 Institute for Pharmacology and Toxicology, Medical Faculty, Martin Luther University Halle-Wittenberg, Magdeburger Straße 4, D-06097 Halle (Saale), Germany; 2 Department of Cardiac Surgery, Mid-German Heart Center, University Hospital Halle, Ernst-Grube-Straße 40, D-06097 Halle (Saale), Germany

**Keywords:** Zacopride, 5-HT_4_, Serotonin receptors, Transgenic mice, Human atrium, Mouse atrium

## Abstract

Zacopride (4-amino-5-chloro-2-methoxy-N-(quinuclidin-3-yl)-benzamide) is a potent agonist in human 5-HT_4_ serotonin receptors in vitro and in the gastrointestinal tract. Zacopride was studied as an antiemetic drug and was intended to treat gastric diseases. Zacopride has been speculated to be useful as an antiarrhythmic agent in the human ventricle by inhibiting cardiac potassium channels. It is unknown whether zacopride is an agonist in human cardiac 5-HT_4_ serotonin receptors. We tested the hypothesis that zacopride stimulates human cardiac atrial 5-HT_4_ serotonin receptors. Zacopride increased the force of contraction and beating rate in isolated atrial preparations from mice with cardiac-specific overexpression of human 5-HT_4_ serotonin receptors (5-HT_4_-TG). However, it was inactive in wild-type mouse hearts (WT). Zacopride was as effective as serotonin in raising the force of contraction and beating rate in atrial preparations of 5-HT_4_-TG. Zacopride raised the force of contraction in human right atrial preparations (HAP) in the absence and presence of the phosphodiesterase III inhibitor cilostamide (1 µM). The positive inotropic effect of zacopride in HAP was attenuated by either 10 µM tropisetron or 1 µM GR125487, both of which are antagonists at 5-HT_4_ serotonin receptors. These data suggest that zacopride is also an agonist at 5-HT_4_ serotonin receptors in the human atrium.

## Introduction

Zacopride falls into the class of substituted benzamides. Its chemical structure is very similar to that of renzapride. Zacopride was developed before 5-HT_4_ serotonin receptors were functionally recognised or cloned. In guinea pig ileal strips, contractions induced by 5-HT were inhibited by zacopride but not by typical 5-HT_3_ antagonists like granisetron (Eglen et al. 1993). This is the first evidence that zacopride acts as a functional partial antagonist at 5-HT_4_ serotonin receptors, an interpretation that is likely because we now know that these 5-HT_4_ serotonin receptors induce contraction in the gastrointestinal tract. Moreover, zacopride also induced contraction, but was less effective than 5-HT and was described as a partial agonist in that model system (Eglen et al. 1993). Similar findings were obtained in the guinea pig colon, where zacopride induced contractions via 5-HT_4_ serotonin receptors (Elswood et al. 1991).

Furthermore, in the model system used to discover 5-HT_4_ serotonin receptors (mouse embryo colliculi neurons), zacopride stimulated adenylate cyclase activity (pEC50-value = 5.95, Dumuis et al. 1989). Zacopride was thus more potent than other benzamides, such as metoclopramide (pEC_50_-value = 5.34), but less potent than the benzamide cisapride (pEC_50_ = 7.14) in this assay (Dumuis et al. 1989). However, zacopride was later used in the context of clinical studies: an oral dose of 400 µg of zacopride was given by endocrinologists; at this dosage, zacopride did not alter blood pressure or heart rate in healthy volunteers (Lefebvre et al. 1993).

Zacopride stimulated the current through L-type calcium channels from isolated atrial human cardiomyocytes, acting as an agonist at 5-HT_4_ serotonin receptors. In contrast, the force of contraction in these atrial preparations has not been studied (Blondel et al. 1997). Zacopride induced dose-dependent tachycardia in anaesthetised pigs (Villalón et al. 1991; Eglen et al. 1993), consistent with a direct stimulatory role of zacopride in the sinus node of the pigs. Moreover, in this model, zacopride is a partial functional agonist: zacopride antagonises the tachycardia induced by 5-HT (Villalon et al. 1991). This motivated us to study the effect of zacopride after serotonin stimulation in isolated human atrial preparations (vide infra).

Elnakish et al. described a negative inotropic effect in the human ventricle at 100 µM of zacopride (Elnakish et al. 2017). Serotonin alone does not increase the force of contraction in human ventricular muscle strips (review: Kaumann and Levy 2006; Neumann et al. 2023a). However, some reports indicate that in the presence of phosphodiesterase inhibitors or end-stage heart failure, serotonin can, via 5-HT_4_ serotonin receptors, increase the force of contraction in the human ventricle (review: Kaumann and Levy 2006).

It is accepted that all inotropic and chronotropic effects of serotonin are mediated via 5-HT_4_ serotonin receptors on human cardiomyocytes (reviews: Kaumann and Levy 2006; Neumann et al. 2017, 2023a). These 5-HT_4_ serotonin receptors are lacking in a functional manner in wild-type mouse hearts: serotonin does not increase the force of contraction in isolated mouse cardiac preparations from wild-type mice (WT, Gergs et al. 2010, 2013). To facilitate the study of human 5-HT_4_ serotonin receptors, we established cardiac-specific overexpression of this receptor in a transgenic mouse (5-HT_4_-TG), which responded with positive inotropic and positive chronotropic effects to agonists (Gergs et al. 2010; review: Neumann et al. 2023a). Hence, we decided to assess whether zacopride would exert positive inotropic and positive chronotropic effects in this 5-HT_4_-TG and not in littermate WT. Furthermore, the effects in 5-HT_4_-TG should be blocked by antagonists at 5-HT_4_ serotonin receptors such as tropisetron or GR125487, and they should be potentiated by a phosphodiesterase inhibitors. One would also expect zacopride to stimulate the 5-HT_4_ serotonin receptors in the human heart and thereby increase the force of contraction.

Hence, we tested the following hypotheses: zacopride increases the force of contraction and beating rate firstly in atrial preparations from 5-HT_4_-TG and secondly in human atrial preparations via 5-HT_4_ serotonin receptors. A progress report has been published in an abstract form (Neumann et al. 2023).

## Materials and methods

### Contractile studies in mice

We used here transgenic mice where the human 5-HT_4_ serotonin receptor is constitutively expressed in the mouse heart by using the α-myosin heavy chain promoter. The generation and initial characterization of these transgenic mice (5-HT_4_-TG) have been reported before (Gergs et al. 2010, 2013). The founder was crossbred over at least four generations with CD-1 mice. The right or left atrial preparations from the mice were isolated and mounted in organ baths under isometric conditions (Gergs et al. 2013; Neumann et al. 1998, 2019). The bathing solution of the organ baths contained 119.8 mM NaCI, 5.4 mM KCI, 1.8 mM CaCl_2_, 1.05 mM MgCl_2_, 0.42 mM NaH_2_PO_4_, 22.6 mM NaHCO_3_, 0.05 mM Na_2_EDTA, 0.28 mM ascorbic acid, and 5.05 mM glucose. The solution was continuously gassed with 95% O_2_ and 5% CO_2_ and maintained at 37 °C and pH 7.4 (Neumann et al. 1998). Spontaneously beating right atrial preparations from mice were used to study any chronotropic effects. Left atrial preparations were stimulated electrically with platinum electrodes with current from a Grass stimulator SD (Ohio, USA). Voltage was direct current and ranged between 5 and 10 Volts, just sufficient to initiate contractions. Electrical impulses had a length of 5 milliseconds. Left atrial preparations allowed us to measure the force of contraction after application of zacopride or other drugs to the organ bath. The signals from the force transducer were electrically amplified, digitized and stored on a commercial personal computer. The signals were measured using a commercial software (Lab Chart 8 from ADInstruments through their distributor Oxford, United Kingdom).

The drug application was as follows: After equilibration was reached, zacopride was cumulatively added to the left atrial or right atrial preparations to establish concentration–response curves. In separate experiments, serotonin was applied cumulatively or first zacopride was applied followed by serotonin. We studied WT and 5-HT_4_-TG from both genders. The average age was 125 days.

### Contractile studies on human preparations

Contractile studies on human preparations were conducted in the same setup and with the same buffer as in the mouse studies (see above). In brief, human right atrial obtained during the cardiac surgery were transferred into the laboratory. Samples were cut and small pieces were mounted under isometric conditions with metal hooks in a glass organ bath. Muscles were electrically stimulated at 1 Hz with rectangular impulses of 5 milliseconds duration and a voltage 10% above threshold for contraction. The force signals amplified and quantified as described above for mouse atrium. The samples were obtained from 16 male patients and two female patients, aged 52–83 years. The patients suffered from coronary heart diseases, two and three vessel diseases, endocarditis, non ST wave elevation myocardial infarction (NSTEMI), atrial fibrillation/flutter and stenosis of the internal carotid artery. Drug therapy included metoprolol, furosemide, apixaban, statins and acetylsalicylic acid. The methods used for atrial contraction studies in human samples have been previously published and were not altered in this study (Gergs et al. 2009, 2017). In this study, we typically gave zacopride cumulatively alone or in the presence of cilostamide. In some experiments, we finally applied antagonists at 5-HT_4_ serotonin receptors (tropisetron, Kaumann et al. 1990, or GR125487, Gergs et al. 2013). In separate experiments, we first applied a single concentration of zacopride and cumulatively applied serotonin or vice versa (see legends). We obtained written informed consent from participating patients.

### Data analysis

Data shown are the means ± standard error of the mean. Statistical significance was estimated using the analysis of variance followed by Bonferroni’s t-test. A p-value < 0.05 was considered significant.

### Drugs and materials

The drugs isoprenaline-hydrochloride, zacopride, serotonin, 5-fluoro-2-methoxy-[1-[2-[(methylsulfonyl)amino]ethyl]-4-piperidinyl]-1*H*-indole-3-methylcarboxylate sulfamate (GR125487), cilostamide, and tropisetron were purchased from Sigma-Aldrich (Taufkirchen, Germany) or Tocris/Bio-Techne (Wiesbaden, Germany). All other chemicals were of the highest purity grade commercially available. Deionised water was used throughout the experiments. Stock solutions were prepared fresh daily.

## Results

**Fig. 1:**
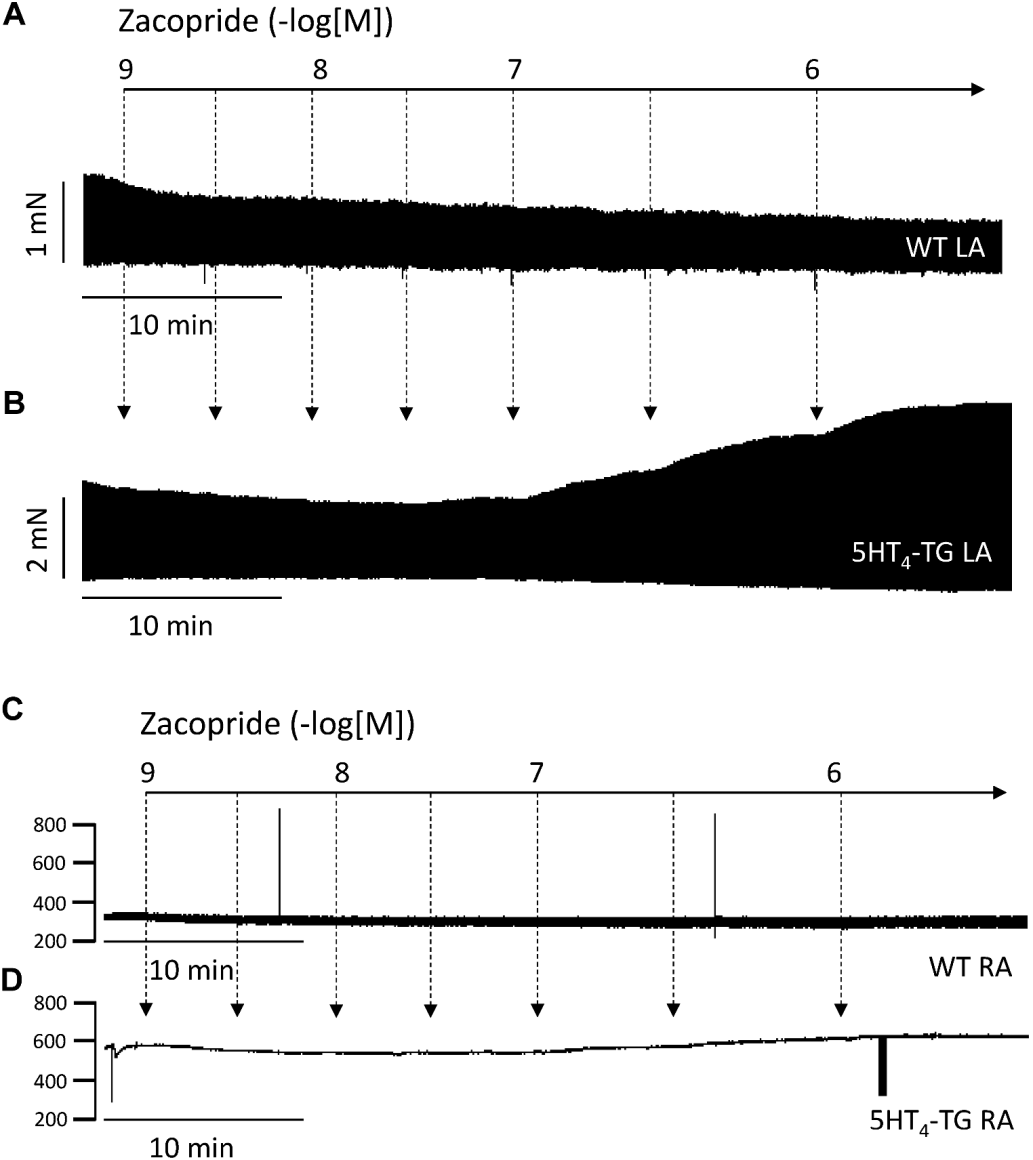
Zacopride induced a time- and concentration-dependent positive inotropic effect in atria from mice with heart specific overexpression of the human 5-HT_4_-receptor (5-HT_4_-TG). **A** Original recordings of force of contraction in isolated electrically stimulated (1 Hz) left atrial preparation from wild type mice (WT) in the presence of increasing concentrations of zacopride. **B** Original recordings of force of contraction in isolated electrically stimulated (1 Hz) left atrial preparation from 5-HT_4_-TG. **C** Original recordings of beating rate in isolated spontaneously beating right atrial preparation from WT. **D** Original recordings in isolated spontaneously beating right atrial preparations from 5-HT_4_-TG. Ordinates indicate force of contraction in milli Newton (mN, Fig. 1A, B) or beats per minute (bpm, Fig. 1C, D). Horizontal bars indicate time in minutes. Abscissae indicate negative logarithmic concentrations of zacopride in the organ bath

Zacopride exerted a concentration- and time-dependent positive inotropic effect in the left atrial preparations from 5-HT_4_-TG (Fig. 1B). In contrast, zacopride failed to raise the force of contraction in the left atrial preparations from WT (Fig. 1A). The latter finding agrees with our previous work: 5-HT cannot raise the force in the atrium from WT (Gergs et al. 2010). The expression or coupling of the receptor is considered to be too small to affect contractility (Gergs et al. 2010). Moreover, zacopride increased the beating rate in spontaneously beating right atrial preparations from 5-HT_4_-TG (Fig. 1D) but not from WT (Fig. 1C).

**Fig. 2:**
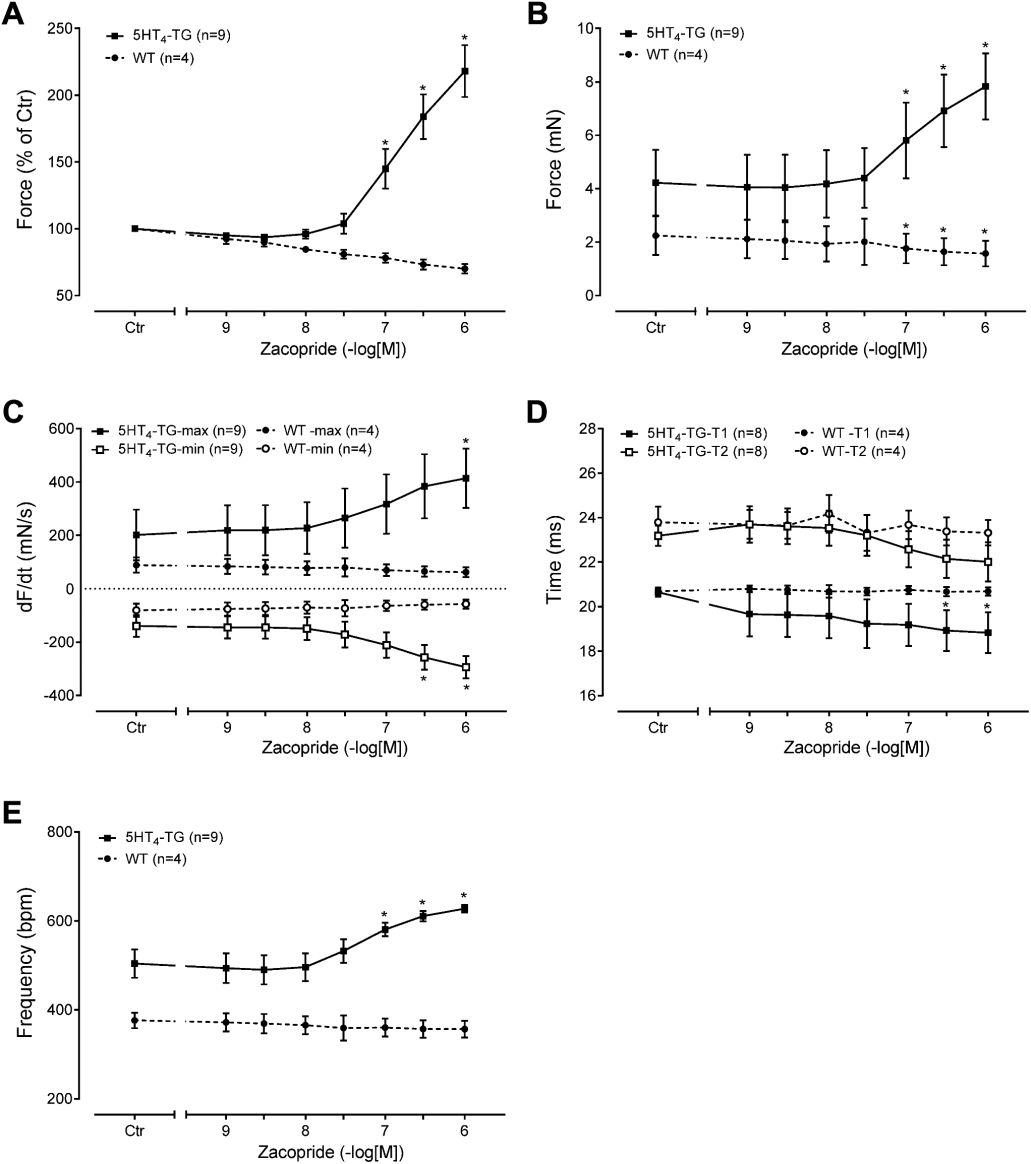
Summarized concentration-response curves for the effect of zacopride on force of contraction in % of pre-drug value (Fig. 2A) or mN (Fig. 2B) or rate of tension development (Fig. 2C) or rate of tension relaxation (Fig. 2C) or time to peak tension (T1, Fig. 2D) or time of relaxation (T2, Fig. 2D) or beating rate (Fig. 2E). * *p* < 0.05 vs. Ctr (pre-drug value). Numbers in brackets mean number of experiments. In Fig. 2C and D, the closed symbols indicate the maximal rate of tension development in 5-HT_4_-TG (squares) and WT (circles). The open symbols refer to the mean the minimum rate of tension development. In Fig. 2D the solid symbols denote the time to peak tension and the open symbols stand for the time of relaxation in 5-HT_4_-TG (squares) and WT (circles). Abscissae indicate negative logarithmic concentrations of zacopride

Several such experiments are summarised concerning the positive inotropic effects if zacopride in percentage of pre-drug value (Fig. 2A) or in absolute force of contraction (Fig. 2B) or with respect to the rate of tension development (Fig. 2C, top). Moreover, zacopride exerted relaxant effects: zacopride increase the rate of muscle relaxation (Fig. 2C, bottom). Moreover, we were interested in the effect of zacopride on the contraction time parameters. These are altered if the 5-HT_4_ serotonin receptors are involved in the contractile effects of zacopride. It turned out that zacopride did not reduce the time to peak tension (Fig. 2D) but reduced the time of muscle relaxation (Fig. 2D). This is expected for agents that increase the phosphorylation of phospholamban. Furthermore, the rate of tension development and the rate of relaxation were augmented in absolute values by zacopride (Fig. 2C).

If zacopride behaves like 5-HT, zacopride should affect the beating rate in the right atrium of 5-HT_4_-TG. Indeed, we noticed concentration-dependent positive chronotropic effect of zacopride as plotted in Fig. 2E.

**Fig. 3:**
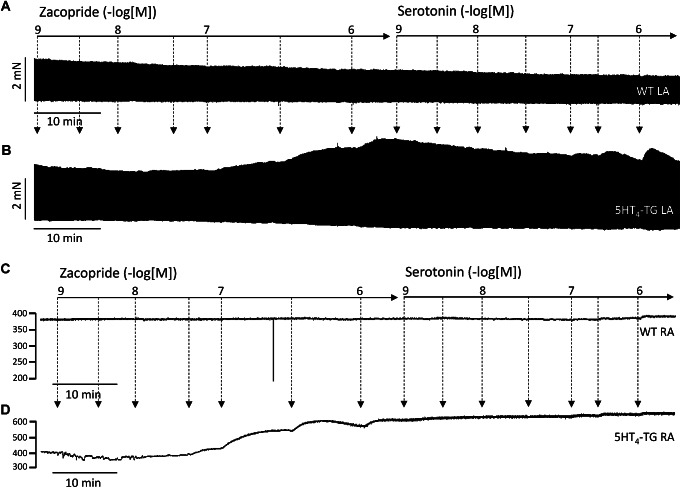
Zacopride is a full agonist in 5-HT_4_-TG.First zacopride was cumulative applied. Thereafter, 5-HT was likewise cumulatively added. Original recordings of force of contraction in isolated electrically stimulated (1 Hz) left atrial preparation from wild type mice (WT) in the presence of increasing concentrations of zacopride and subsequently applied serotonin. Original recording of force of contraction in isolated electrically stimulated (1 Hz) left atrial preparation from 5-HT_4_-TG in the presence of increasing concentrations of zacopride and subsequently applied serotonin. Original recordings of beating rate in isolated spontaneously beating right atrial preparation from WT in the presence of increasing concentrations of zacopride and subsequently applied serotonin. Original recordings in isolated spontaneously beating right atrial preparations from 5-HT_4_-TG in the presence of increasing concentrations of zacopride and subsequently applied serotonin

In separate experiments, we wanted to determine whether zacopride is a full agonist in 5-HT_4_-TG. To this end, we first applied zacopride cumulatively and thereafter we gave serotonin cumulatively. Typical recordings are shown in Fig. 3. Zacopride (1 nM ~ 1 µM) failed to have positive inotropic and chronotropic effects in left and right atria isolated from WT mice, respectively (Fig. 3A C). In left and right atria isolated from 5-HT_4_-TG mice, zacopride increased the spontaneously beating rate and force of contraction, respectively, in a concentration-dependent manner (Fig. 3B and D). Notably, when serotonin was added after zacopride, further increase in the spontaneous rate and force of contraction was not observed (Fig. 3B and D).

**Fig. 4:**
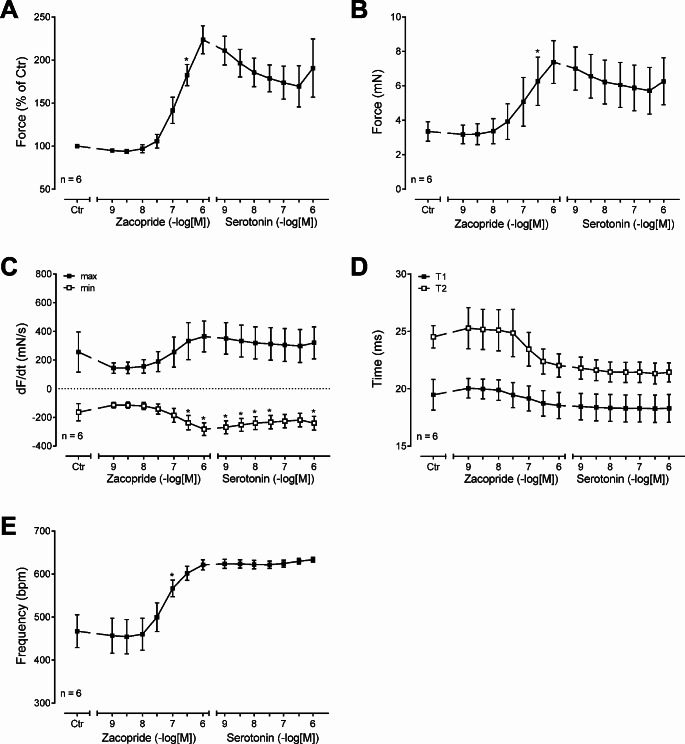
Summarized concentration-response curves for the effect of zacopride and subsequently applied serotonin (for details see Fig. 3) on force of contraction in % of pre-drug value (Fig. 4A) or mN (Fig. 4B) or rate of tension development (Fig. 4C) or rate of relaxation (Fig. 4C) or time to peak tension (T1, Fig. 4D) or time of relaxation (T2, Fig. 4D) or beating rate (Fig. 4E). * *p* < 0.05 vs. Ctr (pre-drug value). Numbers mean number of experiments. Abscissae indicates negative logarithmic concentrations of zacopride. Ordinates indicate force of contraction in % of pre-drug value (Fig. 4A), milli Newton (mN, Fig. 4B), dF/dt in mN per seconds (mN/s, Fig. 4C) or beating rate per minute (bpm, Fig. 4E) or milliseconds (ms)

Such data from several experiments are depicted in summarized in Fig. 4. Here, zacopride increased the force of contraction, and after that, additionally supplied serotonin did not significantly increase the force of contraction any further as measured in % of pre-drug value (Fig. 4A) or in absolute force of contraction in mN (Fig. 4B). Likewise, in left atrial preparations from 5-HT_4_-TG, while zacopride increased the rate of tension development (dF/dt_max_, Fig. 4C) and the rate of relaxation (dF/dt_min_, Fig. 4C), additional serotonin was not significantly more effective than zacopride to raise the rate of tension development (dF/dt_max_, Fig. 4C) and the rate of relaxation (dF/dt_min_, Fig. 4C).

Likewise, while zacopride tended to shorten the time to peak tension and time of relaxation, serotonin did not further reduce these parameters of muscle contraction (Fig. 4D). Finally, whereas zacopride increased the beating rate in the right atrial preparations from 5-HT_4_-TG, additionally applied serotonin could not further stimulate the beating rate, suggesting a similar efficacy of zacopride and serotonin in this transgenic animal model (Fig. 4E).

**Fig. 5:**
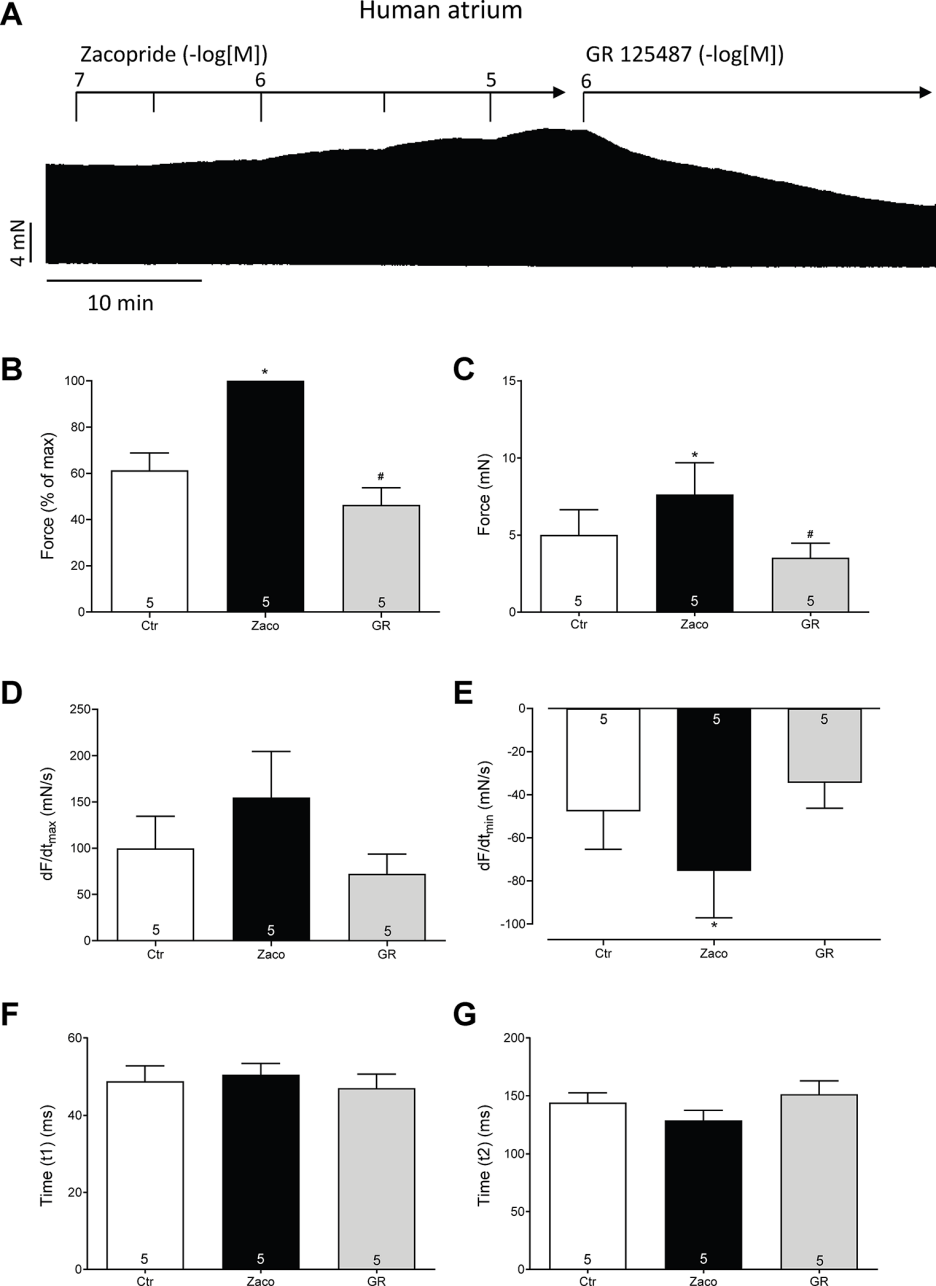
Zacopride is agonist in human atrium. **A** Original recording of the concentration- and time-dependent positive inotropic effect of zacopride in milli Newton (mN) in electrically stimulated human right atrial muscle strips. Horizontal bar indicates time axis in minutes (min). First zacopride (Zaco) was added and then GR 125,487 (GR). Summarized effects of zacopride (10 µM) on force of contraction in % of pre-drug value (Fig. 5B) or mN (Fig. 5C), rate of tension development (Fig. 5D) or rate of relaxation (Fig. 5E) or time to peak tension (Fig. 5F) or time of relaxation (Fig. 5G) or. * *p* < 0.05 vs. Ctr (pre-drug value). # *p* < 0.05 vs. zacopride. Numbers in columns mean number of experiments. The p-values in Fig. 5E amount to Ctr vs. Zaco *p* = 0.024 or Ctr vs. GR *p* = 0.338 or Zaco vs. GR *p* = 0.071

Next, we tested the effects of zacopride on the human heart. To this end, we mounted human atrial preparations in the organ bath, stimulated them electrically, measured isometrically the force of contraction and obtained concentration–response curves for zacopride. As seen in the original recording in Fig. 5A, zacopride time- and concentration-dependently increased the force of contraction in HAP. This increase was attenuated by subsequently applied GR 125,487, an antagonist at 5-HT_4_ serotonin receptors (Fig. 5A). Data for the force of contraction in percent of pre-drug value are summarised in Fig. 5B, and data on force of contraction in absolute values are dipected and 5 C. As with mouse atria, we assessed additional muscle parameters; zacopride tended to increase rate of tension development (Fig. 5D) and augmented the rate of relaxation and this latter effect was attenuated by subsequently applied GR 125,487 (Fig. 5E). Zacopride failed to shorten the time to peak tension (Fig. 5F) and the time of relaxation (Fig. 5G).

**Fig. 6:**
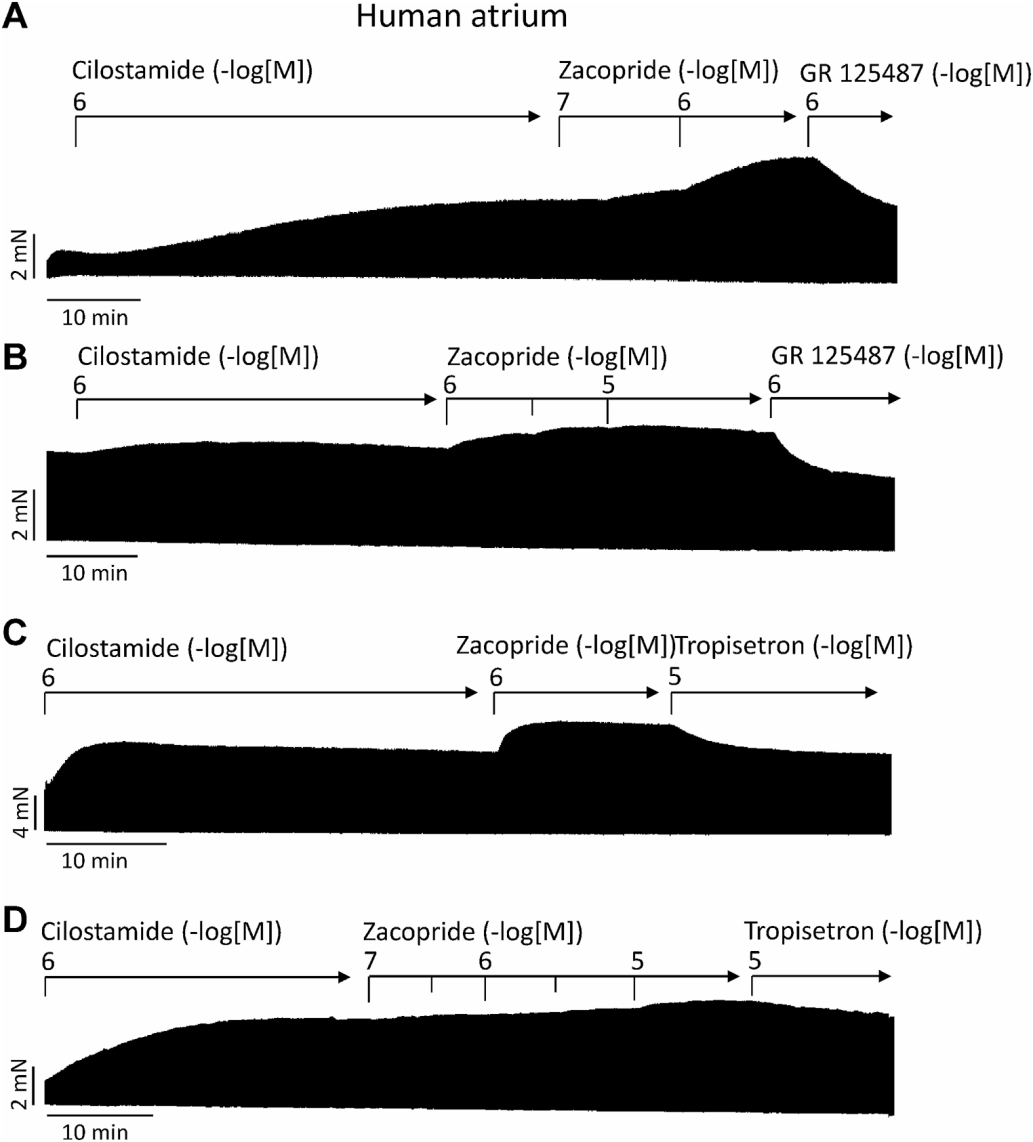
Effects zacopride in the presence of cilostamide (Cilo), a phosphodiesterase inhibitor. Original recordings (experiments from four different patients: Fig. 6A, B, C, D) of the concentration- and time- dependent positive inotropic effect of zacopride (Zaco) in milli Newton (mN) in electrically stimulated human right atrial muscle strips. Horizontal bars indicate time axis in minutes (min). First cilostamide 1 µM was added then 10 µM zacopride and then the 5-HT_4_-receptor antagonists 10 µM tropisetron or 1 µM GR125487.

In addition, zacopride was also able to increase force of contraction in HAP when preparations were first stimulated by cilostamide (an inhibitor of human cardiac-specific phosphodiesterase III) and then we applied zacopride. Under these conditions zacopride also increased force of contraction (depicted in Fig. 6) from several patients and these effects were reversed by tropisetron or GR125487 (Fig. 6). This shows that unlike for lysergic acid diethylamide (Gergs et al. 2024), zacopride in the presence and absence of cilostamide raised force of contraction while lysergic acid diethylamide which was like zacopride an agonist at 5-HT_4_-serotonin receptors, increased force only in the presence and not in the absence of cilostamide (Gergs et al. 2024).

**Fig. 7:**
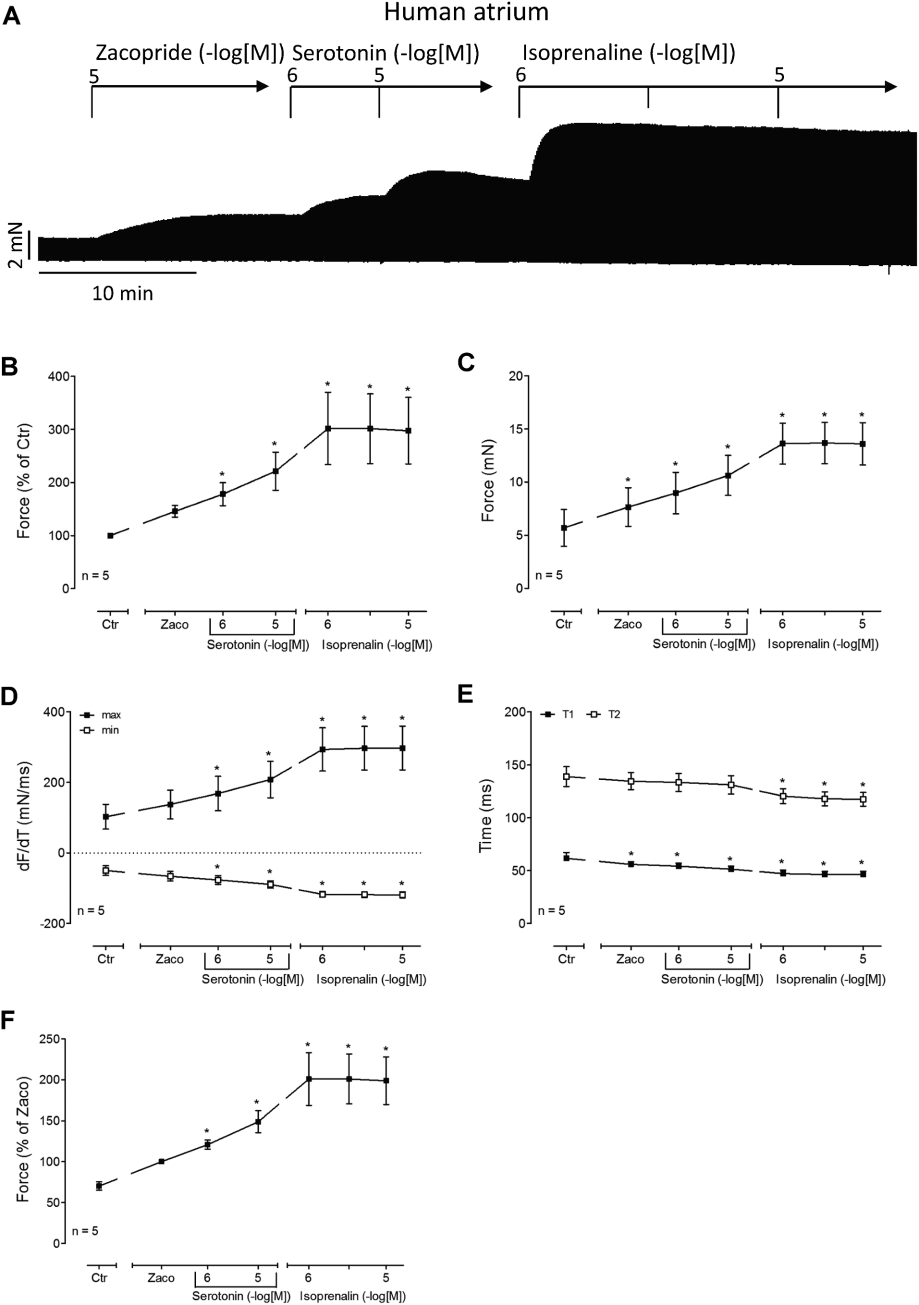
Effects of serotonin in the presence of zacopride. A: Original recording of the concentration- and time- dependent positive inotropic effect of serotonin subsequent to 10 µM zacopride in milli Newton (mN) in electrically stimulated human right atrial muscle strips. Horizontal bar indicates time axis in minutes (min). First 10 µM zacopride and then cumulatively serotonin was added. Summarized effect of serotonin in the presence of zacopride (10 µM) on force of contraction in % of pre-drug value (Fig. 7B) or mN (Fig. 7C) or rate of tension development (Fig. 7D) or rate of relaxation (Fig. 7D) or time to peak tension and time of relaxation (T1, T2, Fig. 7E). * *p* < 0.05 vs. Ctr (pre-drug value). # *p* < 0.05 vs. zacopride (Zaco). Numbers mean number of experiments. Abscissae indicates negative logarithmic concentrations of zacopride. Ordinate in Fig. 7A in mN, in Fig. 7B in % of pre-drug value and in Fig. 7C in milli seconds (ms). Rate of contraction and rate of relaxation in Fig. 7D in mN/ms. Time to peak tension and time to relaxation in ms (Fig. 7E). Ordinate in Fig. 7F, give force when the effect of zacopride on force of contraction was defined as 100%. Then the effects of additionally applied serotonin and isoprenaline on force of contraction were calculated based on this value for zacopride (Fig. 7F). Abscissae indicate molar concentrations of zacopride or serotonin or isoprenaline in negative logarithms. Significant difference versus control (Ctr; pre-drug value) is indicated with asterisks. Numbers in brackets mean number of experiments

To understand whether zacopride is a partial agonist in the isolated human atrium, we devised a different protocol. We first gave zacopride to raise force of contraction and then added two concentrations of serotonin, finally we added isoprenaline concentration dependently. A typical original experiment is displayed in Fig. 7A. Noticeable is the positive inotropic effect of zacopride. Thereafter, serotonin elevated the force of contraction further. The muscle however, was not maximally stimulated under these conditions, because additionally supplied isoprenaline could raise force of contraction further Fig. 7A. This was quantified for force of contraction in percent of the pre-drug value (Fig. 7B), for the absolute force (Fig. 7C), for the rate of tension development, and for the rate of relaxation (Fig. 7D). Moreover, zacopride reduced the time to peak tension but failed to diminish the time of relaxation (Fig. 7E). Additionally applied isoprenaline reduced thereafter time of relaxation (Fig. 7E). Zacopride alone shortened time to peak tension but not time of relaxation (Fig. 7E). Additionally, applied serotonin failed to alter time to peak tension further (Fig. 7E). An alternative way to plot these data was used in Fig. 7F. Here, we arbitrarily set the effect of zacopride on force of contraction at 100%. Then additionally applied serotonin and isoprenaline increased force (Fig. 7F).

**Fig. 8:**
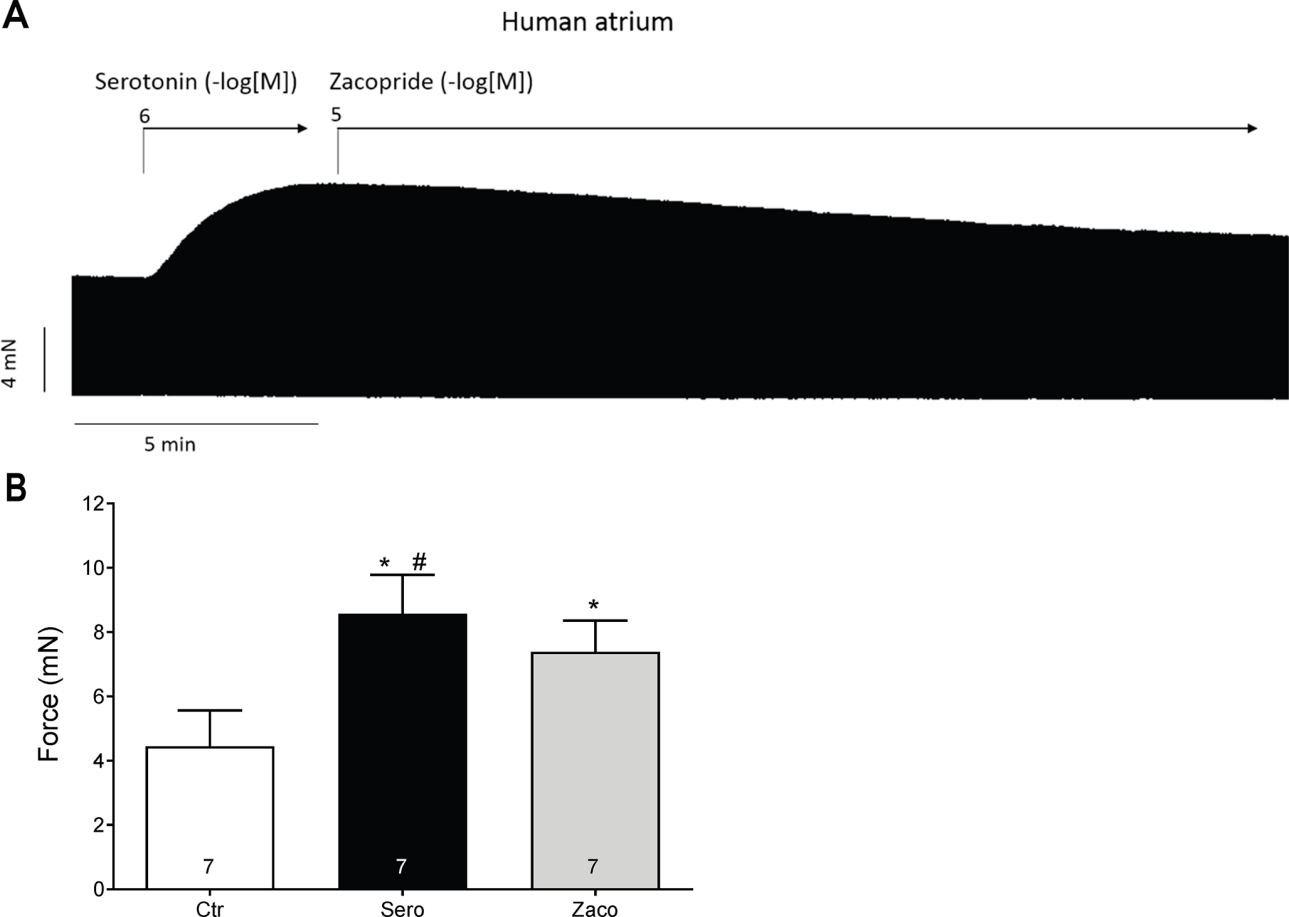
Effects of zacopride in the presence of serotonin. A: Original recording of the time-dependent negative inotropic effect of zacopride after serotonin in milli Newton (mN) in electrically stimulated human right atrial muscle strips. Horizontal bar indicates time axis in minutes (min). First serotonin and then zacopride were applied as a single dose. Summarized effect of zacopride (10 µM) after the serotonin (1 µM) on force of contraction in mN (Fig. 8B). * *p* < 0.05 vs. Ctr (pre-drug value). # *p* < 0.05 vs. 10 µM zacopride. Ordinates in Fig. 8A and B are in mN. Numbers in columns mean number of experiments

In further experiments, as depicted in Fig. 8A, we first raised the force of contraction using 1 µM serotonin, then we applied additionally zacopride 10 µM (Fig. 8A). Under these conditions, the zacopride reduced the force of contraction previously raised using serotonin (Fig. 8A). This negative inotropic effect of zacopride was probably not due general damage of the muscle by zacopride because subsequently applied isoprenaline was able to elevate force of contraction further (Fig. 8A) Such data for the force of contraction in mN are summarised in Fig. 8B.

## Discussion

### Primary new findings

The primary finding of this study is that zacopride can function as a partial agonist at human 5-HT_4_ serotonin receptors in the beating heart. Zacopride is a full agonist in transgenic mice, which expresses the human 5-HT_4_ serotonin receptor (5-HT_4_-TG) and a partial agonist in the isolated human right atrium via human 5-HT_4_ serotonin receptors. We explain these differences by the much higher 5-HT_4_ serotonin receptor expression density in the atrium of 5-HT_4_-TG than in the human atrium.

### Mechanism of zacopride

We suggest that zacopride increased force and beating rate as an agonist at cardiac human 5-HT_4_ serotonin receptors because zacopride only increased contractility in the atrium from 5-HT4-TG and not in WT. By comparing the concentration–response curves of zacopride to those of serotonin in atrial preparations, one can conclude that zacopride at 5-HT_4_ serotonin receptors in the left and right atrium acts as a full agonist; serotonin was no more effective than zacopride. This holds for the force of contraction (left atrium) and the beating rate (right atrium).

Zacopride acts as an agonist at 5-HT_4_ serotonin receptors in the isolated human atrium. This effect was blocked by tropisetron, acting here as a 5-HT_4_ serotonin antagonist (Kaumann et al. 1990) and GR145487, a selective 5-HT_4_ serotonin receptor antagonist. Hence, we tentatively concluded that the in vivo effects of zacopride in the human heart on the force of contraction are mediated by 5-HT_4_ serotonin receptors.

A partial agonist is an agonist which is unable to induce full activation of a receptor. A partial agonist competitively inhibits the effects of the full agonists, which makes them to act either as a functional agonist or a functional antagonist. In Fig. 8A, the addition of 10 µM zacopride can reduce the positive inotropic effect of 1 µM serotonin. In Fig. 7A, however, serotonin (1 and 10 µM) can evidently increase force of contraction even in the presence of 10 µM zacopride. Hence, at least a concentration (10 µM) where zacopride acts as full agonist in left atrial preparations from 5-HT_4_-TG, in the human heart (with lower expression of 5-HT_4_ serotonin receptors: Hesse et al. 2024), zacopride is not a full functional agonist. In HAP, 10 µM zacopride slightly reduced force of contraction that was fully stimulated by 1 µM serotonin, suggesting functional antagonism.

One could ask why others noted a negative inotropic effect of zacopride in isolated human ventricular preparations (Elnakish et al. 2017). One must remember that this effect was in the human ventricle, where 5-HT alone does not increase the force of contraction. Hence, a lack of a positive inotropic effect in the human ventricle is not unexpected. One might speculate that, at 100 µM, zacopride (the only concentration that reduced force in their study) has an unspecific deleterious effect on cardiac proteins in mitochondria (Elnakish et al. 2017). However, our data agree with previous studies from other labs and ours that 5-HT increases the force of contraction in the human atrium. Other agonists at 5-HT_4_ serotonin receptors, such as cisapride, prucalopride, or metoclopramide, increase the force of contraction in the human atrium (Kaumann et al. 1990; Gergs et al. 2009; Chai et al. 2012).

Moreover, it is noteworthy that zacopride alone increases the force of contraction in the human heart. We have recently reported that there are partial agonists at 5-HT_4_ serotonin receptors, such as lysergic acid diethylamide and ergotamine, that increase the force of contraction in the human atrium only in the presence of the phosphodiesterase III inhibitor cilostamide (Gergs et al. 2024; Jacob et al. 2023).

### Role of phosphorylation of regulatory proteins

**Fig. 9:**
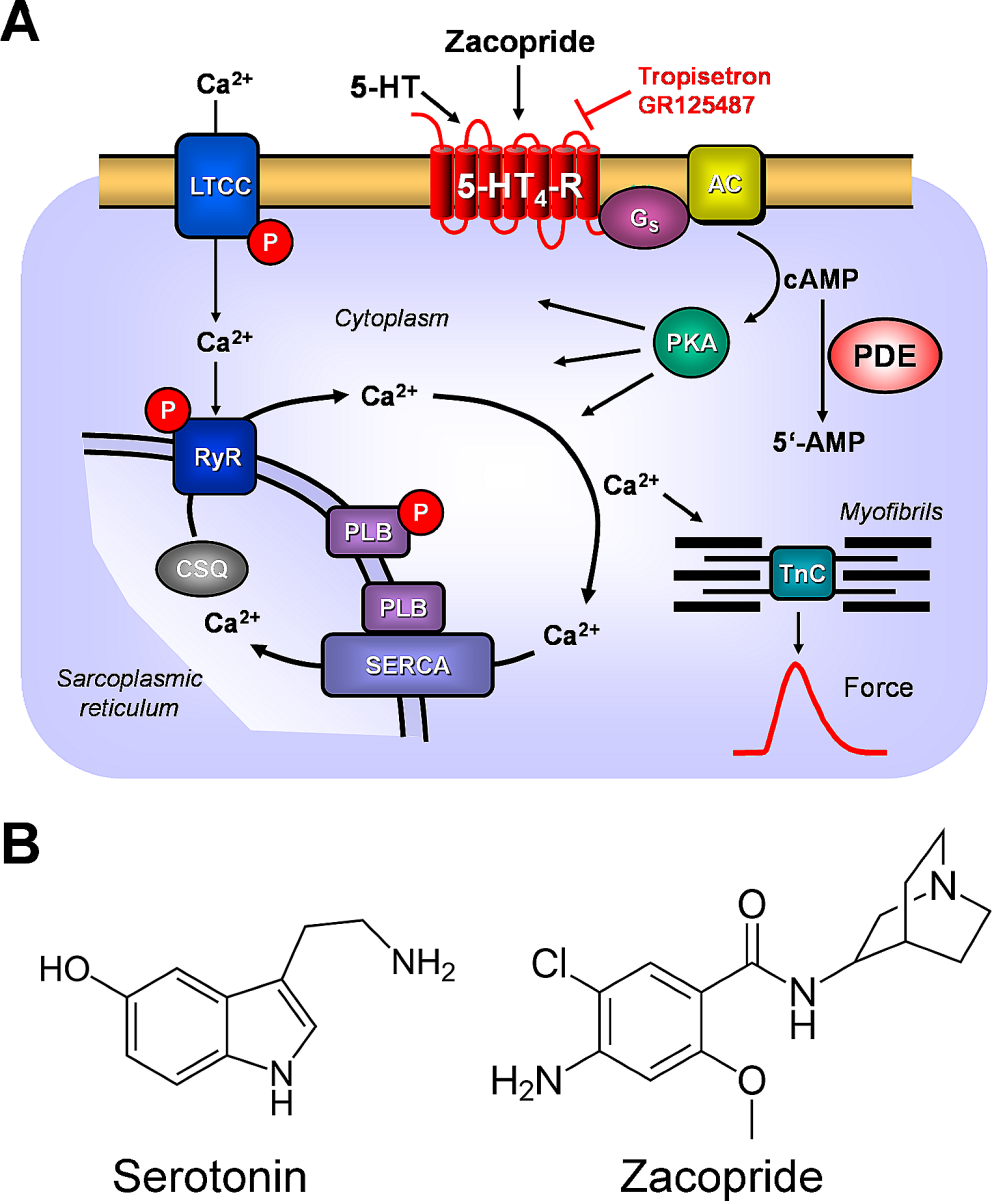
**A** (Scheme): Mechanism(s) of action of serotonin and zacopride in cardiomyocytes. A heptahelical 5-HT_4_-serotonin receptor is depicted in sarcolemma. The agonist serotonin (5-HT) activates the 5-HT_4_-serotonin receptor. Thereby, the stimulatory G-protein (G_s_) augments the ability of adenylyl cyclases (AC) to generate cAMP. This cAMP can activate cAMP-dependent protein kinases (PKA). Thereafter, PKA phosphorylates and activates target proteins like the L-type Calcium channel (LTCC) in the sarcolemma and the ryanodine receptor (RyR) in the sarcoplasmic reticulum (SR). Phosphorylation of phospholamban increases the activity of SR-Ca ATPAse (SERCA). Phosphodiesterase (PDE) III converts cAMP to inactive 5´-AMP in the human heart. Tegaserod may activate human cardiac 5-HT_4_-serotonin receptors. **B** Structural formulae of serotonin and zacopride. Note the benzamide structure in zacopride, the different side chain of zacopride compared to serotonin and the optical center in zacopride. We used the racemic zacopride

The general assumption is that 5-HT_4_ serotonin receptor stimulation increases the phosphorylation of protein substrates for cAMP-dependent protein kinase (Fig. 9). Others and we have found that serotonin via 5-HT_4_ serotonin receptors can increase the phosphorylation state of phospholamban. This phosphorylation might explain the lusitropic effects of zacopride in the human atrium. Moreover, the reduction in the rate of relaxation also argues for the role of the action of zacopride on potassium channels in the human atrium. If zacopride stimulated this IKr greatly, as in rats (Kii and Iso 1997), this is expected to lead per se to a shorting of the time for muscle contraction.

### Species differences

Notably, zacopride acted more potently and effectively and as a full agonist to raise the force in transgenic mice (5-HT_4_-TG) than in the human atrium. This is consistent with our previous work on cisapride, prucalopride, or metoclopramide (Keller et al. 2018; Neumann et al. 2021. We assume this is due to the much higher level of expression of 5-HT_4_ serotonin receptors in mouse hearts than in human hearts. There is evidence for this at least for other receptors and we would argue the same might be true for 5HT_4_-serotonin receptors. For instance, inducible overexpression in cell culture on D_2_-dopamine receptors revealed that higher expression led to a more potent action of a partial agonist, aripiprazole, at this receptor (Koener et al. 2012). We ourselves noted that when A_1_-adenosine receptor density increased, this altered even the signal transduction mechanism and the functional role (force of contraction) of the A_1_-adenosine receptors in transgenic mice (Neumann et al. 1999). We believe that the 5-HT_4_-TG offer the possibility of amplifying any effect of agonists at 5-HT_4_ serotonin receptors. On the other hand, if a putative agonist does not act in 5-HT_4_-TG, this agonist is unlikely to work as an agonist in human tissue.

Another species difference is worth noting. Previous work on the cardioprotective role of zacopride was performed in rats. However, in the literature, in healthy rats, 5-HT raises the force of contraction in the rat atrium via 5-HT_2A_ serotonin receptors (Läer et al. 1998). Hence, the beneficial effects of zacopride in rat ischaemia models are probably not translatable to the clinic. Similar studies in 5-HT_4_-TG on the protective effects of zacopride against ischaemia might be more meaningful, but were beyond the scope of this study.

### Effects on the beating rate

Next, we discuss our findings in the mouse right atrial preparations. We assume that, like 5-HT, zacopride also stimulated 5-HT_4_ serotonin receptors in the mouse heart (5-HT_4_-TG). This conclusion is based on the observation that the effect is absent in the right atrium from the WT. The zacopride acted like various other agonists (cisapride, prucalopride, and metoclopramide) as an agonist on 5-HT_4_ serotonin receptors in the sinus node (Keller et al. 2018; Neumann et al. 2021). This observation is potentially relevant because there is no easy way to study the chronotropic effect of 5-HT_4_ serotonin receptor stimulation. Typically, the sinus node is not touched in cardiac surgery and, hence, is not the subject of many studies. However, in our 5-HT_4_-TG, we might in the future study whether zacopride has a proarrhythmic effect like 5-HT (Keller et al. 2018) or whether the antiarrhythmic effect of zacopride prevails, which would be relevant to future clinical studies with zacopride.

### Limitations of the study

One can argue that we have not assessed the effects on the sinus node of man directly. Such a study would require access to a human pacemaker. Such studies were beyond the scope of this initial study. We did not have the opportunity to study contractility in human ventricle tissue due to a lack of access to that tissue. However, the expression and inotropic function of 5-HT_4_ serotonin receptors are increased in patients with end-stage heart failure (Afzal et al. 2008; Brattelid et al. 2004). Hence, might be reasonable to assume that in the failing human ventricle, zacopride has a positive inotropic effect. However, the opposite was the case. Elnakish et al. (2017) have studied the effect of zacopride in muscle ventricular strips in the organ bath from failing and non-failing human hearts. They did not detect a positive inotropic effect of zacopride: neither in failing nor in non-failing ventricular samples. They even reported that 100 µM zacopride exerted a negative inotropic effect on human ventricular preparations. They speculated that detrimental actions of zacopride on mitochondrial function could explain the negative inotropic effects of zacopride in isolated human ventricular preparations (Elnakish et al. 2017).

Moreover, zacopride exists as an R-zacopride and S-zacopride. These enantiomers exhibit different affinities for 5-HT_4_ serotonin receptors in binding studies (Eglen et al. 1994; Ge et al. 1997). However, we chose for this initial report to use only racemic zacopride, in order to facilitate comparison of our data with the work of others in cardiac preparations that also used racemic zacopride (e.g. Ouadid et al. 1992; Elnakish et al. 2017; Kii and Ito 1997; Sun et al. 2017; Lin et al. 2020; Liu et al. 2019, 2021).

In summary, we can now address the hypotheses raised in the Introduction: Zacopride raised the force of contraction and beating rate in 5-HT_4_-TG and elevated the force of contraction in the human heart via 5-HT_4_ serotonin receptors.

## Data Availability

The data in this study are available from the corresponding author upon reasonable request.
